# Hierarchical Catalysts Prepared by Interzeolite Transformation

**DOI:** 10.1021/jacs.2c00665

**Published:** 2022-03-10

**Authors:** Monica J. Mendoza-Castro, Erika De Oliveira-Jardim, Nelcari-Trinidad Ramírez-Marquez, Carlos-Alexander Trujillo, Noemi Linares, Javier García-Martínez

**Affiliations:** † Laboratorio de Nanotecnología Molecular, Departamento de Química Inorgánica, Universidad de Alicante, Ctra. San Vicente-Alicante s/n, 03690 Alicante, Spain; ‡ Laboratorio de Catálisis Heterogénea, Departamento de Química, Universidad Nacional de Colombia, Carrera 45 # 26-95, 111321 Bogotá, Colombia

## Abstract

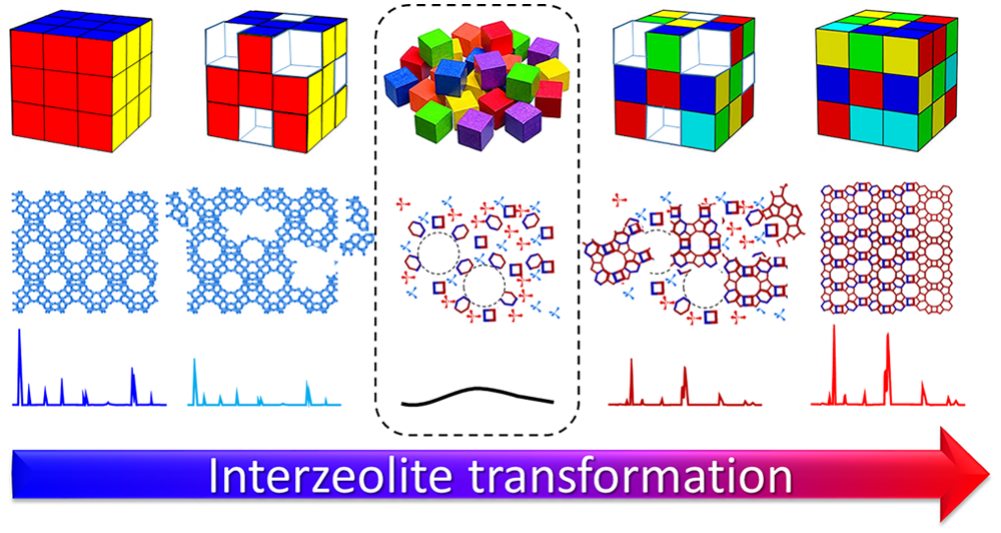

Interzeolite transformation has been used to produce a novel family of hierarchical catalysts featuring excellent textural properties, strong acidity, and superior catalytic performance for the Friedel–Crafts alkylation of indole with benzhydrol, the Claisen–Schmidt condensation of benzaldehyde and hydroxyacetophenone, and the cracking of polystyrene. Intermediate solids of the FAU interzeolite transformation into BEA display both increased accessibility—due to the development of mesoporosity—and strong acidity—caused by the presence of ultrasmall crystals or zeolitic fragments in their structure. The use of surfactants allows for the development of the hierarchical catalysts with very narrow pore size distribution. The properties of interzeolite transformation intermediates (ITIs) can be fine-tuned simply by stopping the interconversion at different times.

## 1 Introduction

For more than five decades, zeolites have been used as catalysts in some of the most important industrial processes due to their combination of strong acidity, well-defined structure, and excellent hydrothermal stability.^1,2^ Today, worldwide consumption of zeolite catalysts reaches ∼250000 metric tons per year.^3^ Their commercial impact is difficult to overestimate; they are responsible, for example, for ca. 30% of the gasoline produced in refinery operations.^4^ These crystalline microporous materials are typically prepared under hydrothermal conditions from amorphous aluminosilicate gels in the presence of structure-directing agents (SDAs), which can be inorganic cations and/or organic molecules, in basic or fluoride media. Zeolites can also be produced by the densification of other more open structures,^5^ a process usually referred to as zeolite interconversion or interzeolite transformation.^6^ This method is a convenient strategy for the synthesis of many relevant structures, often requiring shorter synthesis times than traditional approaches.^6^ However, although the interzeolite transformation is a widely used and versatile approach, its mechanism remains elusive.^7^

On the other hand, despite their pivotal role in catalysis and their wide use in separation and adsorption, the intrinsic microporosity of zeolites imposes severe diffusion limitations to large-sized reactant/product molecules,^8,9^ limiting conversion, while increasing coke formation, which results in an accelerated catalyst deactivation. Two main approaches are used to improve mass transport in zeolites: (i) the preparation of hierarchical zeolites featuring a secondary mesoporous system^10−12^ and (ii) the generation of nanosized crystals.^13^

By combining these two approaches, mesoporous solids composed of zeolite fragments or nanocrystals were prepared. To achieve this objective, these two strategies were used: the first is the synthesis of mesoporous solids using zeolitic precursors, as performed by Pinnavaia and co-workers, who reported the formation of mesoporous materials using FAU, MFI, and *BEA zeolites seeds.^14−16^ These materials presented higher catalytic activity for the transformation of bulky molecules and improved hydrothermal stability than equivalent mesoporous solids prepared from noncrystalline precursors, which was attributed to the presence of zeolitic fragments. The second strategy is based on the use of organic SDAs and/or the partial crystallization of zeolites, starting from the typical synthetic conditions of zeolite formation and quenching the synthesis in an initial amorphous phase.^17−23^ This method produced materials with superior catalytic performance as compared to the fully crystalline materials for the conversion of bulky molecules and, usually, improved hydrothermal stabilities over mesoporous amorphous solids.

Recently, a new approach has been added to the toolbox by Valtchev and co-workers: embryonic zeolites, which are ultrasmall zeolite crystals supported on an aluminosilicate matrix.^24−27^ They combine high specific surface area and a micro-/mesoporous architecture (1–3 nm), resulting in excellent accessibility and enhanced catalytic performance in the dealkylation of triisopropylbenzene or the synthesis of dimethyl ether from syngas.

Herein, we present a new strategy for the synthesis of superior hierarchical catalysts, whose properties evolve during interzeolite transformation. They are composed of zeolitic fragments and display improved accessibility. Because of these features, they effectively catalyze reactions involving large molecules. We realized this strategy for the interconversion of FAU into BEA. Additionally, we used quaternary ammonium surfactants to develop well-defined mesoporosity in the intermediates. By stopping the interconversion of FAU into BEA at different times, we were able to produce Interzeolite Transformation Intermediates (ITIs) showing optimized catalytic performance for the Friedel–Crafts alkylation of indole and the Claisen–Schmidt condensation of benzaldehyde.^28−30^ Similarly, these catalysts also significantly reduce the cracking temperature of polystyrene, a model reaction for the assessment of the catalytic performance of cracking catalysts.^31,32^

## 2 Methods

### Materials

USY zeolite (CBV720) and BEA zeolite (CP814E) were supplied by Zeolyst, with a nominal Si/Al ratio of 15 and 12.5, respectively. The following chemicals were purchased from Sigma-Aldrich (St. Louis, MO): tetraethylammonium hydroxide aqueous solution (35 wt % fraction), cetyltrimethylammonium bromide (CTAB), indole (99%), benzhydrol (>99%), 2′-hydroxyacetophenone, benzaldehyde, and polystyerene (*M*_w_ = 35000). Sodium hydroxide (98% pellets) was supplied by Fluka.

### FAU to BEA Interzeolite Transformations

**Scheme 1:**
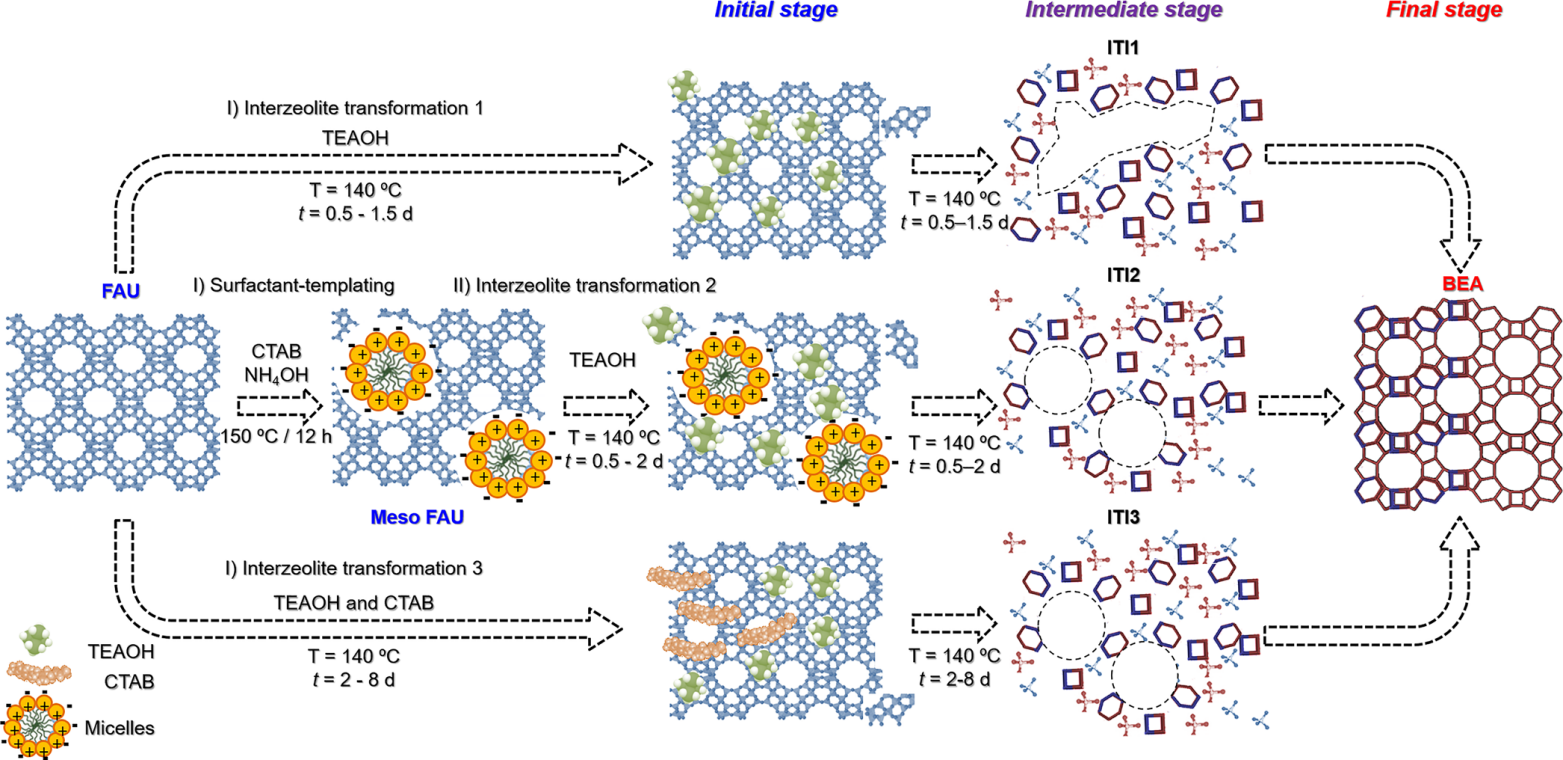
Schematic Representation of the Three Interzeolite Transformation Methods Evaluated The parent zeolite (FAU) was hydrothermally treated (ITI1) in the presence of TEAOH, (ITI2) the same procedure by starting from an uncalcined surfactant-templated FAU zeolite, and (ITI3) similar to ITI1 by using a mixture of TEAOH and CTAB. The treatments were maintained until fully crystalline BEA zeolite was obtained.

To obtain mesoporous intermediates with improved textural properties, three different methods were evaluated for the transformation of FAU zeolite into BEA (see Scheme 1). In the first method, the original microporous CBV720 (FAU) zeolite was interconverted into beta zeolite (BEA) by using tetraethylammonium hydroxide (TEAOH) as SDA. One gram of CBV720 zeolite was stirred in a Teflon-lined stainless-steel autoclave with 2 mL of a 4.3 M aqueous TEAOH solution for 1 h; then, the mixture was hydrothermally treated at 140 °C for 0.5–1.5 days under static conditions. The obtained material was filtered, washed with distillated water, dried at 60 °C overnight, and calcined at 550 °C for 5 h (2 °C min^–1^). The hierarchical materials prepared by using TEAOH as SDA (Interzeolite Transformation Intermediates 1) were labeled as ITI1-*x*, where *x* is the time of treatment in days.

In a second approach, a mesoporous FAU zeolite prepared by surfactant templating^33^ was used as starting material in the interzeolite transformation into BEA. The surfactant-templated FAU zeolite was obtained by using the method described elsewhere.^33^ In brief, 2.5 g of the commercial FAU zeolite (CBV720) was mixed with 157 mL of a basic surfactant solution ([NH_4_OH] = 0.19 M; CTAB = 0.5 g); the mixture was then stirred at room temperature for 20 min. The synthetic mixture was transferred to a Teflon-lined stainless-steel autoclave where the hydrothermal treatment was performed at 150 °C for 12 h under static conditions. The sample was filtered, washed, and dried at 60 °C for 12 h. Finally, 1.2 g of the uncalcined surfactant-templated FAU zeolite was interconverted into BEA by using the procedure described in ITI1 for 0.5–2 days. The materials prepared by using this strategy were labeled as ITI2-*x*, where *x* is the time of treatment in days.

Third, the combined use of a quaternary ammonium surfactant (CTAB) and a SDA (TEAOH) was explored. This procedure was adapted from ref (34). In a typical synthesis, 1 g of CVB720 (FAU) zeolite was stirred in 2 mL of an aqueous solution of CTAB (0.6 M) and TEAOH (4.3 M) for 1 h. The mixture was transferred to a Teflon-lined stainless steel autoclave and statically heated at 140 °C for 2–8 days. The products were filtered, washed, and dried at 60 °C overnight. Finally, they were calcined at 550 °C for 5 h (2 °C min^–1^) to remove both the surfactant and SDA. The materials prepared by using this treatment were named ITI3-*x*, where *x* is the time of treatment in days.

### Materials Characterization

X-ray diffraction (XRD) patterns were obtained in a powder X-ray diffractometer (Bruker AXS D8 Advance) with graphite monochromatized Cu Kα radiation at 40 kV and 40 mA. A known amount of graphite (used as internal standard) was mixed with the samples before the analysis to quantify the crystallinity of the different solids. The most intense peak of graphite ((002) at 26° 2θ) was used to normalize all the spectra. The most crystalline BEA zeolite obtained after interzeolite transformation is completed, this is, zeolite ITI1-1.5, was defined as 100% crystallinity and used as reference to calculate the percentage of BEA in the ITI samples. Nitrogen physisorption isotherms at −196 °C were performed in an AUTOSORB-6 apparatus. The samples were previously degassed for 8 h at 250 °C at 5 × 10^–5^ bar. Adsorption data were analyzed by using the software QuadraWinTM (ver. 6.0) of Quantachrome Instruments. Cumulative pore volumes and pore-size distribution curves were calculated by using the DFT method (NL-DFT adsorption branch model). The total pore volume was obtained at the plateau of the cumulative adsorption pore volume plot at a relative pressure (*P*/*P*_0_) of 0.9. Micropore volume was determined by NL-DFT as the volume adsorbed at pore sizes <2 nm, and the mesopore volume was calculated by subtracting the micropore volume from the total pore volume as shown in ref (12). High-resolution argon physisorption isotherms at −196 °C and low P/P_0_ range (10^−7^–0.01) were performed in an ASAP 2425 apparatus from Micromeritics. The morphology of the samples was evaluated by transmission electron microscopy (TEM) images collected by using a JEM-1400 Plus microscope (JEOL, 120 kV, 0.38 nm resolution). The UV-Raman spectra were recorded on a Jasco NRS-5100 dispersive Raman system with a laser source of 325 nm. The composition of the prepared solids was determined by X-ray fluorescence (XRF) in a sequential spectrometer PHILIPS MAGIX PRO equipped with a rhodium X-ray tube and beryllium window. The total number of Brønsted acid sites in the samples was measured by isopropylamine decomposition in a simultaneous thermal analyzer (Linseis HP-STA) as described in ref (35) and in the Supporting Information.

### Catalytic Evaluation

The Friedel–Crafts alkylation reactions were performed as follows: ca. 0.04 mmol of substrate (5 mg of indole) was stirred with 26 mg of benzhydrol (diphenylmethanol, 0.14 mmol) in a glass vessel containing 5 mg of the catalyst. The vials were closed and transferred into an aluminum heating block preheated to 80 °C. The mixture was stirred (400 rpm) at this temperature for 30 min.

Claisen–Schmidt condensation reactions were performed in a similar manner by mixing 10 mg of catalyst, 1.4 mmol of benzaldehyde (142.7 μL), and 0.7 mmol of hydroxyacetophenone (85 μL) at 135 °C for 48 h.

To ensure reproducibility, three replicate experiments were conducted both for Friedel–Crafts alkylation and for Claisen–Schmidt condensation reactions. After reaction, the mixtures were cooled, washed with acetone, and centrifuged. Products were identified by gas chromatography coupled to mass spectrometry (GC-MS). The conversion was calculated by calibration of the indole and hydroxyacetopenone (GC-FID) as limiting reactants for the Friedel–Crafts and Claisen–Schmidt reactions, respectively. Turnover frequencies (TOFs) were calculated as moles of indole or hydroxyacetophenone converted after 30 min and 48 h of reaction, respectively, per mole of total acid sites.

The catalytic cracking of polystyrene was performed in a NETZSCH TGA/STA 449 F5 Júpiter thermogravimetric analyzer. The reactions were conducted by loading and evenly spreading 1 mg of the catalyst in the platinum microcrucible prior to the addition of 10 mg of the polymer. The samples were heated under nitrogen flow (100 mL min^–1^) from 70 to 700 °C at 10 °C min^–1^. The temperature of the maximum degradation rate (*T*_max_) for every experiment was determined from the derivative thermogravimetric (DTG) plots. Duplicate experiments, conducted for selected samples, produced deviations below ±1 °C. The apparent TOF was calculated by dividing the moles of polymer decomposed at 350 °C per minute by moles of total acid sites (see the Supporting Information for more details).

## 3 Results

### Evolution of the Structural and Textural Properties

**Figure 1:**
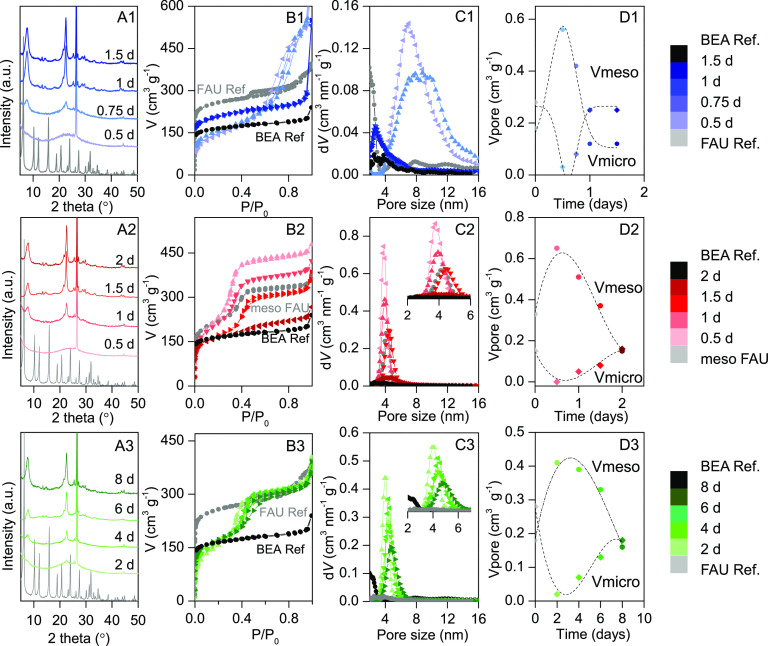
Structural and textural characterization of the samples obtained at different times using the three interzeolite transformation methods (1, top, blue), ITI1, (2, center, red), ITI2, and (3, bottom, green), ITI3. (A) XRD patterns of the products obtained at different treatment times. (B) N_2_ physisorption isotherms at −196 °C of the same products and (C) their corresponding NL-DFT pore size distributions. (D) Micropore (diamonds) and mesopore (circles) volumes of the samples produced by each method at different times. The color codes are shown in the legend (right).

The solids recovered at different times during the interzeolite transformation of FAU into BEA were thoroughly characterized to gain insight into how their properties evolve as one zeolite (FAU) interconverts into the other (BEA). Three different approaches were explored (summarized in Scheme 1): (1) CBV720 was hydrothermally treated in the presence of TEAOH (ITI1), (2) an uncalcined surfactant-templated CBV720 was also treated with TEAOH (ITI2), and (3) CBV720 was treated with a mixture of TEAOH and CTAB (ITI3). The treatment was maintained until no more BEA zeolite was obtained. A time-resolved study for each one of the three approaches was conducted by interrupting the interconversion at different treatment times to monitor the evolution of the properties of the ITI materials, see Table S1, which includes a summary of all the synthesized samples and their characterization. The development of the BEA phase was followed by powder XRD analysis (Figure 1(A1–A3)). Under the conditions used, interzeolite transformation yields amorphous solids at short times. As observed in Figure 1(A1–A3), FAU zeolite completely loses its crystallinity before BEA zeolite starts to form. Interzeolite transformation through an amorphous phase has been reported elsewhere.^36^ The authors postulated that FAU zeolite is interconverted into BEA zeolite via a liquid-phase-mediated mechanism. The alkaline media produces the amorphization and partial dissolution of the parent FAU zeolite; however, as it will be discussed later on, these mesoporous intermediates are composed of FAU fragments that evolve to produce BEA structural units. The evolution of the Si/Al ratio of the solids over time supports this liquid-mediated mechanism (Figure S1). The amorphous material presents the lowest Si/Al ratio of all the ITI samples, yet the solids recovery was very high in all cases (>50%, see Table S1) because of the presence of the quaternary amines, which inhibit desilication. As the interconversion continues, the Si/Al ratio almost recovers its initial value, indicating the incorporation of the dissolved material into the final zeolite. It is worth mentioning that this increase in the Si/Al ratio of the samples coincides with the formation of the BEA zeolite (see Figure S1), as evidenced by X-ray diffraction data (Figure 1(A1–A3)). The bump in the baseline at ca. 25° 2θ at short times of transformation indicates the presence of a residual amount of amorphous phase in the sample. Fully crystalline BEA zeolite was obtained by using any of the three methods, if enough time is allowed. In all cases, the bump of the baseline gradually disappears. Even though the structural evolution of the samples is very similar in the three methods, different kinetics are observed (Figures 1 and 3E). ITI1 and ITI2 have very similar kinetics; however, the presence of the surfactant inside the FAU zeolite slightly delays its interconversion (ca. 50% of BEA phase in 24 h instead of 18 h, Figure 3E). However, the addition of the CTAB to the transformation mixture (ITI3) significantly slows down the conversion. When this method is used, the first XRD peaks due to the BEA phase appear only after 2 days of hydrothermal treatment (see Figure 1(A3)). As shown in Figure 3E, a 50% of BEA crystallinity is reached after 6 days of treatment; this is 8 times longer than when using ITI1. This marked delay is likely due to the presence of surfactant in the reaction mixture, which can interfere with the crystallization process. The competition between the two quaternary amines used in the ITI3 method causes a significant delay in the interzeolite transformation, which is driven by the TEAOH. However, in the case of the ITI2, the presence of CTAB only in the interior of the FAU zeolite barely impacts the kinetics of the interzeolite transformation (Figure 3E). We performed a control experiment to determine the effect of CTAB inside the zeolite in the kinetics of the transformation. A calcined surfactant-templated FAU zeolite was transformed by using the ITI1 method, yielding very similar materials and kinetics than when using a conventional FAU zeolite (CBV720) and the same ITI1 method, as shown in Figures S2 and S3 (see the Supporting Information for additional details). This confirms that the embedded surfactant only slightly delays the interzeolite transformation process.

The N_2_ physisorption isotherms of all samples are shown in Figure 1(B1–B3), and their corresponding pore size distribution (PSD) in Figure 1(C1–C3). The parent FAU zeolite (CBV720) shows a type I isotherm, which is typical of microporous materials. There is a continuous uptake of N_2_ from *P*/*P*_0_ > 0.8 due to the porosity generated by the supplier during the ultrastabilization of the zeolite by steaming.^37^ The intermediate materials can be divided in two groups: (1) those obtained by using the ITI1 method, which displays a broad N_2_ uptake at a relative pressure ca. *P*/*P*_0_ = 0.7, indicating the presence of large and irregular mesopores, and (2) those obtained by ITI2 and ITI3 methods which present a sharp N_2_ uptake at ca. *P*/*P*_0_ = 0.3, characteristic of surfactant-templated materials. From the XRD and N_2_ physisorption data we conclude that the parent FAU structure undergoes through an amorphous mesoporous phase before the BEA zeolite forms (see also Figure 1(A1–A3)). This amorphous phase is also evidenced by the very low microporous volume of the intermediate samples (Figure 1(D1–D3)). At longer times of transformation, the obtained samples show very different textural properties. Mesoporosity disappears as the new BEA microporous phase emerges. This evolution is clearly shown in Figure 1(D1–D3). With regard to the type and size of the pores, in the case of the FAU zeolite treated with TEAOH (ITI1) the intermediate samples show a wide distribution of mesoporosity (Figure 1(C1)) ranging from 4 to 16 nm, centered at around 8 nm. On the other hand, the incorporation of CTAB in the interzeolite transformation, either by using a noncalcined surfactant-templated zeolite (ITI2) or by adding CTAB to the reaction mixture (ITI3), yields materials with much narrower PSD (see Figure 1(C2,C3)) and a smaller average pore size ca. 4 nm, which is in good agreement with the size of the CTAB micelle.^38^ The incorporation of surfactants in the interzeolite transformation by means of any of the above-mentioned methods allows for the precise modulation of the textural properties of those ITIs and for the development of a well-defined and tunable mesoporosity.

A deeper analysis of the transformation from FAU to BEA zeolite was performed by monitoring the changes of the micropore structure of the solids by Ar physisorption at −196 °C. As shown in Figure S4, FAU and BEA zeolites present very distinct isotherms in the low *P*/*P*_0_ region (10^–7^ to 0.01) due to the different size, shape, and connectivity of their micropore systems. Interestingly, the adsorption profile of the intermediate samples evolves from that of the FAU zeolite to that of the BEA zeolite. This observation indicates that the intermediates do not possess a bimodal micropore system, but rather this evolves from the microporous structure of the parent FAU zeolite to that of BEA zeolite. At low *P*/*P*_0_, the isotherms of the samples produced at short times of treatment resemble that of the mesoporous Al-MCM-41 material, indicating their amorphous mesoporous character and confirming the conclusions obtained by XRD. However, at longer treatment times, the new micropore system of the samples becomes increasingly similar to that of BEA zeolites. The study of the evolution of the isotherms at low *P*/*P*_0_ provides detailed information about the changes in the microporous structure of solids, which is especially useful in the case of interzeolite transformation and complementary to the information obtained by XRD data.

**Figure 2:**
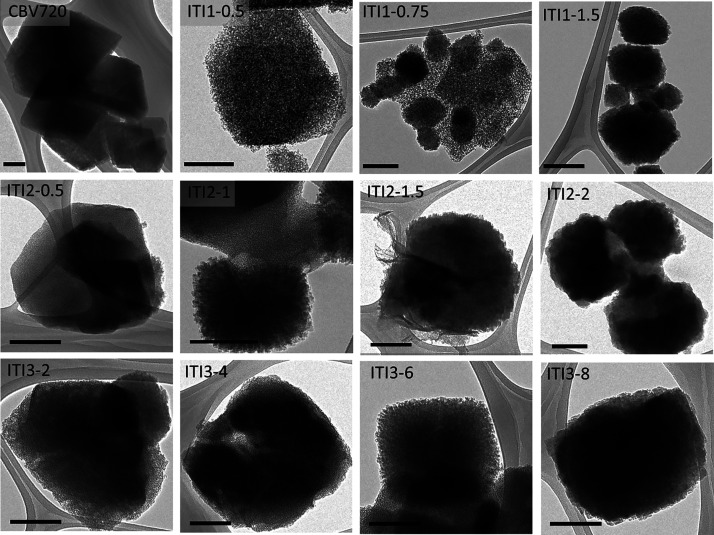
TEM micrographs of the products obtained at different times by using the three interzeolite transformation methods: (top) ITI1, (center) ITI2, and (bottom) ITI3. The names of the samples, indicating the treatment times, are included in each micrograph. Scale bar represents 200 nm.

The changes in the morphology and microstructure of the solids during interzeolite transformation were further studied by TEM (Figure 2). A mesoporous sponge-like material, which maintains the initial shape of the FAU crystals, was obtained during the initial stages of hydrothermal treatment, indicating that some desilication or partial dissolution occurs, which is consistent with the decrease in the Si/Al ratio of the samples at short times of treatment as aforementioned. This porosity is highly homogeneous when the surfactant is present during the interzeolite transformation (ITI2 and ITI3). As treatment progresses, BEA nanocrystals start to form on the surface of the amorphous phase (see Figure 2, images ITI1-0.75, ITI2-0.5, and ITI3-4. They grow in size and population over time to finally yield only fully crystallized BEA zeolite. As evidenced by TEM, the interconversion of FAU into BEA undergoes through an amorphous phase, from which BEA nanocrystals develop. As described by Ivanova et al.,^39^ interzeolite transformations can proceed through two mechanisms: (1) a solid → solid transformation (from amorphous to crystalline) and (2) a solution-mediated mechanism involving the dissolution of the initial phase. On the basis of our data, we speculate that a combination of these two methods occurs during the transformation of FAU into BEA. First, part of the original zeolite dissolves, as evidenced by the reduction of the Si/Al ratio (XRF) and TEM studies. The structural units of the new zeolite form from these fragments. In fact, as it has been described elsewhere,^40^ the formation of 5-membered rings (5R) units, present in BEA structure, from a zeolite which does not contain this kind of unit, such as the FAU structure, which is made of 6- and 4-membered rings, should occur through dissolved silicate species from a medium- or high-silica zeolite. The new zeolite grows from the mesoporous amorphous phase, as zeolite nanocrystals (see Figure 2, images ITI1-0.75, ITI2-0.5, and ITI3-4). The growth of the BEA zeolite from these nanocrystals proceeds via a solid–solid mechanism (as high recovery yield was obtained in all cases). A nonclassical mechanism of crystal growth can also be involved, as it has been described for the crystallization of zeolites from amorphous precursors.^41^ The intimate contact of the different phases suggests the assembly and attachment of fragments or particles from the amorphous phase to the new BEA phase. From all these results, we conclude that at one point of the interzeolite transformation highly mesoporous materials containing immature crystals of BEA are formed. They constitute a whole family of new materials, which properties can be kinetically adjusted by simply interrupting the interzeolite transformation at different times, allowing for the development of optimized catalysts for the transformation of bulky molecules.^25^

**Figure 3:**
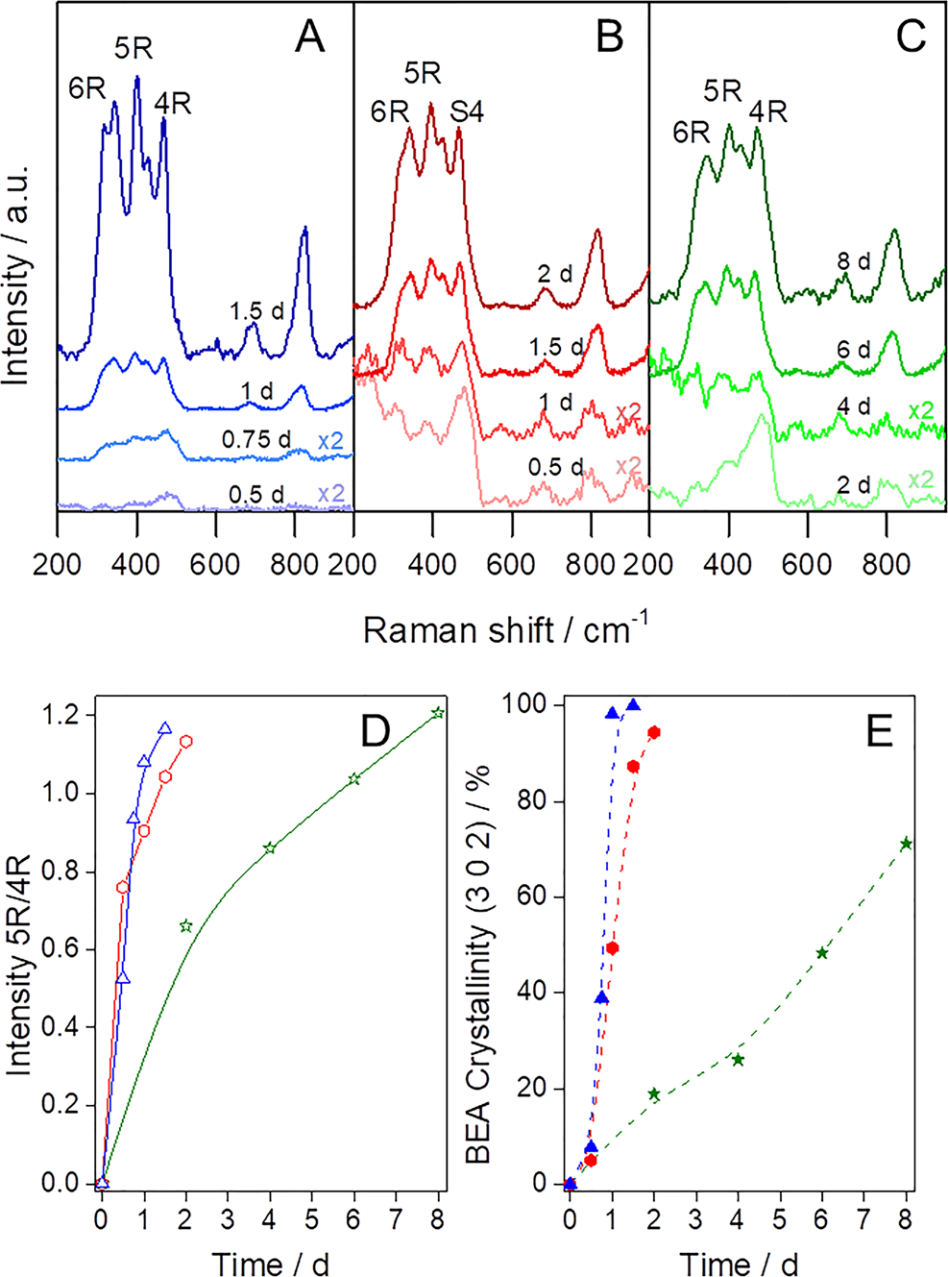
Time-resolved UV-Raman spectra of samples prepared by using the interzeolite transformation method: (A) ITI1, blue; (B) ITI2, red; and (C) ITI3, green. The treatment times are indicated over each spectrum. Evolution of the (D) ratio between the intensities of the 5R and 4R bands in UV-Raman and (E) BEA crystallinity, calculated against the intensity of the (302) peak of the most crystalline sample (ITI1-1.5).

The structural evolution of the samples was further analyzed by UV-Raman spectroscopy.^42^Figure 3A–C shows the time-resolved UV-Raman spectra of solids prepared by the three different methods. The initial FAU zeolite (see Figure S5) shows the typical three bands associated with its structural units: (i) a strong band at ca. 508 cm^–1^ for the breathing mode vibration of the 4-membered ring (4R),^43^ (ii) a shoulder band at 490 cm^–1^ due to the breathing vibration mode of the 4R in the 6-membered double rings (D6R),^44^ and (iii) a third band at ca. 300 cm^–1^ corresponding to the bending mode of the D6R.^43,45^ In the initial stages of the transformation, the characteristic band of the D6R units almost disappears, which confirms the loss of the FAU structure (Figure 1(A1–A3)); however, a very small band attributed to the 6-membered rings (6Rs) formed by decomposition of the D6R structures can be observed in the UV-Raman spectra.^44^ In all cases, the band due to the 4R members is quite noticeable, even though much less prominent. Simultaneously, the 4R bands shift to lower values, indicating that the 4Rs units in the FAU structure (ca. 500 cm^–1^) gradually transform into 4Rs units in the BEA structure (ca. 465 cm^–1^).^44^ The presence of these 4R and 6R units in the solids since the beginning of the transformation indicates that these shared units do not completely disappear during the process; instead, they evolve from the FAU to the BEA framework. Moreover, their existence in the ITI samples can be related to the formation of mesoporous material-containing zeolite building units, which is usually associated with higher hydrothermal stability and stronger acidity as compared to amorphous aluminosilicate mesoporous materials that do not show these features.^16,46^ At longer treatment times, the bands attributed to the 6Rs (at 320 and 345 cm^–1^) increase, while an incipient band at 400 cm^–1^ starts to develop due to the formation of the 5R units. The 5R units are associated with the formation of BEA fragments in the hierarchical catalysts. FTIR analyses show the same structural evolution (see more details in Figure S6).

Figure 3D shows the evolution over time of the ratio of the intensities of the band corresponding to 5R units (at ca. 400 cm^–1^), which are only present in BEA zeolite, and that corresponding to 4R units (at ca. 500–465 cm^–1^), which are present in both FAU and BEA zeolites. Therefore, this ratio (5R/4R) can be used as a proxy for the amount of BEA zeolite per total amount of zeolite. Therefore, its evolution can be used to monitor the interzeolite transformation (Figure 3D). Although the XRD patterns do not show any noticeable BEA peaks because of the poor long-range crystallinity (Figure 3E), the 5R bands in the Raman spectra, even at early stages of the interzeolite transformation, confirm the presence of BEA units (small fragments) in the hierarchical catalysts.

### Catalytic Evaluation of the Samples

**Figure 4:**
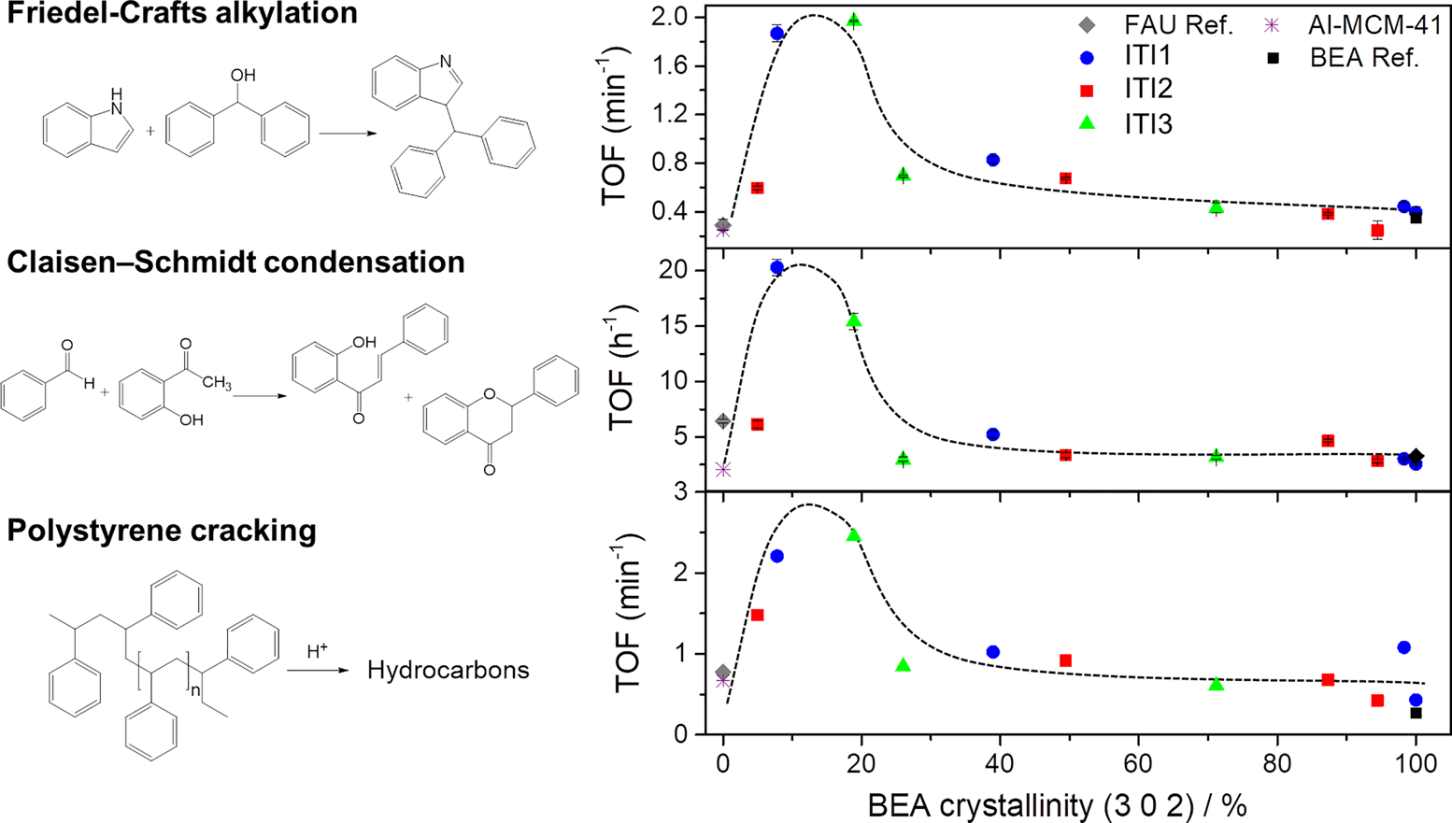
Catalytic performance (TOF) of the ITI materials for the (top) Friedel–Crafts alkylation of indole with benzhydrol, (center) Claisen–Schmidt condensation of benzaldehyde and hydroxyacetophenone, and (bottom) polystyrene cracking as a function of the degree of BEA transformation, expressed as BEA crystallinity.

The parent zeolites and the solids produced by interzeolite transformation were tested for three different reactions, which are widely used to assess the catalytic performance of mesoporous zeolites.^29−32^ First, the catalysts were tested in two fine-chemistry reactions producing very large derivatives, which formation might be inhibited by the narrow porosity of the zeolites, namely, the Friedel–Crafts alkylation of indole with benzhydrol and the Claisen–Schmidt condensation of benzaldehyde and hydroxyacetophenone (see Figure 4). Second, the catalysts were evaluated in the cracking of polystyrene, a bulky compound whose accessibility in microporous zeolites is greatly hindered (Figure 4).

With regard to the Friedel–Crafts alkylation, in all cases, the intermediate materials, which feature high mesoporosity and medium acidity, exhibited higher conversion than both the parent FAU zeolite and the microporous BEA zeolite (final product of the interzeolite transformation; see Figure 4 (top) and Table S2). The improved accessibility of the hierarchical catalysts resulted in a 6-fold increase in TOF over the commercial FAU (CBV720, parent zeolite). Similarly, the commercial BEA zeolite (CP814E) shows lower catalytic activity than the materials prepared by interzeolite conversion. These results confirm the well-known role of mesoporosity in the production of bulky compounds. However, an Al–MCM-41 with similar Si/Al ratio (Table S2) also shows significantly lower conversion, indicating that mesoporosity alone is not enough and that strong acidity is also needed. Analogous results were obtained for the Claisen–Schmidt condensation reaction. By plotting the turnover frequency of the catalysts for both reactions as a function of the BEA crystallinity (used here as a proxy of the degree of interzeolite transformation), we were able to identify that the best performing catalysts were produced during the early intermediate stages of the FAU to BEA transformation (ca. 15% BEA crystallinity). These catalysts have low–medium acidity and high–medium mesoporosity, as shown in Figure S7. As reported elsewhere,^47,48^ both accessibility and strong acidity are needed in the heterogeneous catalytic transformation of fine chemicals, as large substrates and/or intermediate products are usually involved and the reaction typically takes place in liquid phase.^49−51^ Consequently, conventional zeolites (FAU and BEA) and the amorphous mesoporous solid (Al–MCM-41) display the lowest catalytic activity (Figure 4 and Figure S7).

We also tested our materials for the transformation of very bulky starting compounds, namely, the catalytic cracking of polystyrene. In this case, the activity of each catalyst was related to its capacity to lower the temperature of maximum degradation rate. As can be observed in Table S3 and Figure S8, the ITI materials reduce, in all cases, the temperature of degradation of polystyrene up to a maximum of ca. 60 °C as compared to the parent CBV720 zeolite. This reduction was higher for the materials with more mesoporosity; however, as aforementioned for the Friedel–Crafts alkylation and Claisen–Schmidt condensation reactions, mesoporosity is not sufficient, as an Al–MCM-41 with a similar Si/Al ratio was not able to shift the degradation temperature to the same degree (38 °C vs 60 °C). The presence of strong acidity, provided by the zeolite fragments present in the intermediates, is also needed to achieve optimum performance. The apparent TOF of each catalyst was obtained at constant temperature (350 °C), as shown in Figure S8. It is quite remarkable that also in the case of the cracking of polystyrene (Figure 4, bottom) the best performing catalysts were the same ones that provided optimum results for the Friedel–Crafts alkylation and the Claisen–Schmidt condensations reactions. This is, those obtained when the interzeolite transformation reached ca. 15% BEA crystallinity. The striking similarity of the curves shown in Figure 4 obtained for quite different reactions, reactants, and conditions not only confirms the superior performance of the hierarchical catalysts prepared by partial zeolite interconversion but also highlights the possibility of fine-tuning their behavior simply by stopping the interconversion at different times.

## 4 Conclusions

Interzeolites Transformation Intermediates (ITIs) are excellent hierarchical catalysts whose properties evolve as one zeolite transforms into another. These intermediates yield superior catalytic performance in reactions involving bulky molecules due to their improved accessibility and strong acidity. The use of CTAB in the synthesis (either by embedding it inside a surfactant-templated zeolite or by its addition to the reaction mixture) allows for the precise control of the textural properties of the intermediates, resulting in materials with very narrow pore size distribution. Intermediates with well-defined mesoporosity and BEA nanocrystals in their structure show a 6-fold increase in TOF in the Friedel–Crafts alkylation of indole using a bulky alcohol as alkylating agent than the parent FAU zeolite. Similar results were obtained in the Claisen–Schmidt condensation. In this case, the best performing hierarchical catalysts displayed a 3-fold enhancement in TOF over the parent FAU. As controls, the commercial BEA and a mesoporous Al-MCM-41 with similar Si/Al ratio both present lower catalytic performances, confirming the key role of both mesoporosity and acidity to carry out these reactions. Similar results were obtained in the cracking of polystyrene, confirming the superior catalytic performance of the hierarchical catalysts prepared by interzeolite transformation in the conversion of bulky molecules. An important advantage of this strategy is that the physicochemical properties and, therefore the catalytic performance, of the hierarchical catalysts can be finely tuned by simply stopping the interzeolite transformation at different times. This creates countless opportunities for the development of hierarchical catalysts with optimized properties and superior catalytic performance for those reactions in which zeolites present significant diffusion limitations.

## Supporting Information Available

Experimental details regarding the catalytic evaluation of the materials, supplementary tables (with the description of the samples and the catalytic results) and figures (showing the Si/Al ratio, UV-Raman and FTIR spectra of the discussed samples) (PDF)
