# Correction to “Hierarchical Catalysts Prepared by Interzeolite Transformation”

**DOI:** 10.1021/jacs.2c03298

**Published:** 2022-04-14

**Authors:** Monica J. Mendoza-Castro, Erika De Oliveira-Jardim, Nelcari-Trinidad Ramírez-Marquez, Carlos-Alexander Trujillo, Noemi Linares, Javier García-Martínez

Page 5168. In Figure 4, the Friedel–Crafts alkylation reaction contained a typographical error. The corrected figure is presented here.

**Figure 4:**
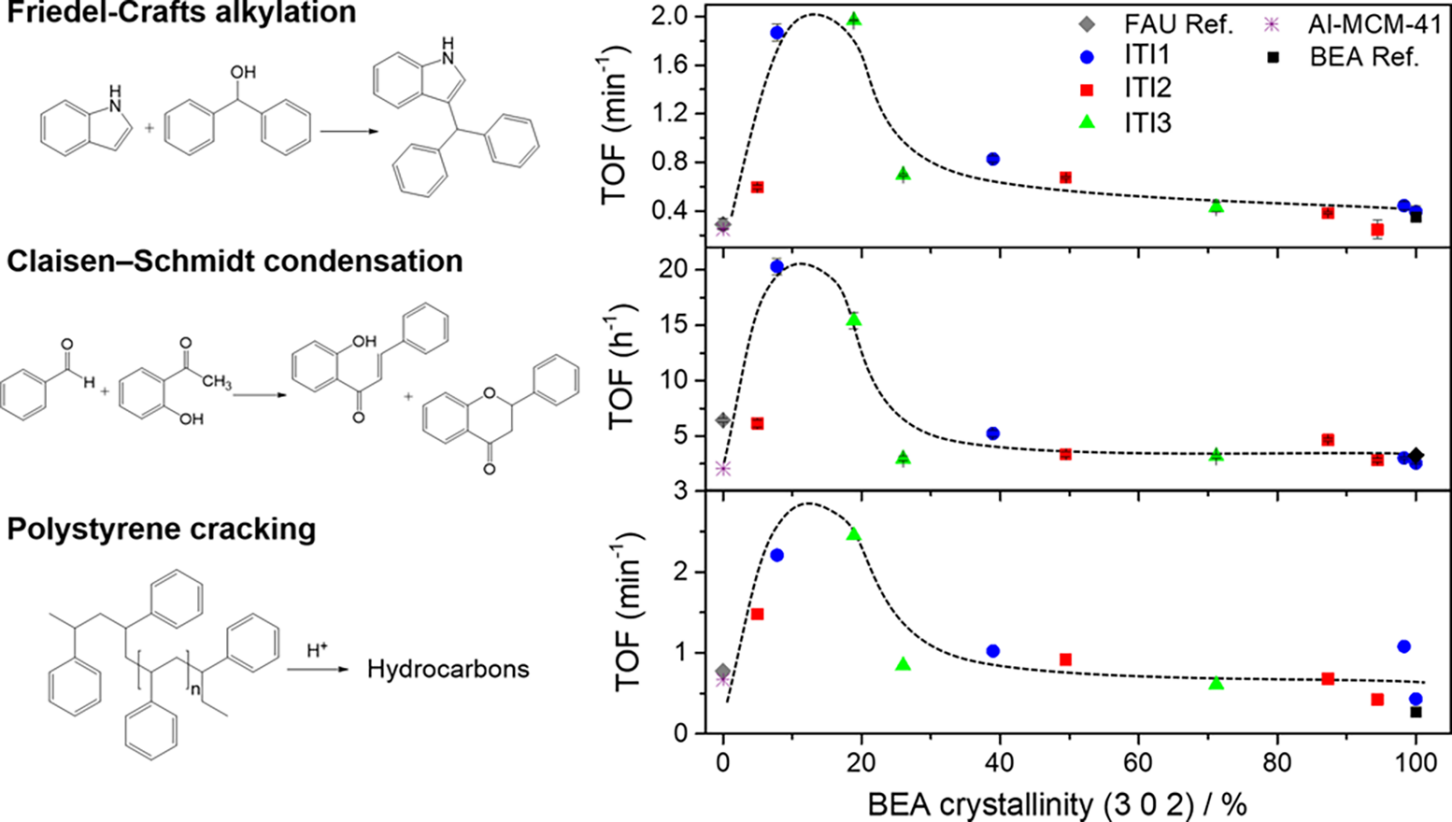
Catalytic performance (TOF) of the ITI materials for the (top) Friedel–Crafts alkylation of indole with benzhydrol, (center) Claisen–Schmidt condensation of benzaldehyde and hydroxyacetophenone, and (bottom) polystyrene cracking as a function of the degree of BEA transformation, expressed as BEA crystallinity.

