# Supplementary material for: Hierarchical Catalysts Prepared by Interzeolite Transformation
Source: J Am Chem Soc. 2022 Mar 10;144(11):5163–71. doi: 10.1021/jacs.2c00665 (PMC8949765; doi:10.1021/jacs.2c00665)
Supplement: Supplementary file 1 — ja2c00665_si_001.pdf [file ja2c00665_si_001.pdf]

Hierarchical catalysts prepared by interzeolite transformation

Monica J. Mendoza-Castro[a], Erika De Oliveira-Jardim[a,†], Nelcari-Trinidad Ramírez-Marquez[b], Carlos-Alexander Trujillo[b], Noemi Linares[a,*], Javier García-Martínez[a,*]

[a] Laboratorio de Nanotecnología Molecular, Departamento de Química Inorgánica, Universidad de Alicante, Ctra. San Vicente-Alicante s/n, 03690 Alicante, Spain.

[b] Laboratorio de Catálisis Heterogénea, Departamento de Química, Universidad Nacional de Colombia, Bogotá, Colombia.

KEYWORDS. Hierarchical catalysts, interzeolite transformation, surfactant-templating, enhanced accessibility, improved performance

ABSTRACT: Interzeolite transformation has been used to produce a novel family of hierarchical catalysts featuring excellent textural properties, strong acidity, and superior catalytic performance for the Friedel-Crafts alkylation of indole with bezhydrol, the Claisen–Schmidt condensation of benzaldehyde and hydroxyacetophenone, and the cracking of polystyrene. Intermediate solids of the FAU interzeolite transformation into BEA display both increased accessibility – due to the development of mesoporosity – and strong acidity – caused by the presence of ultrasmall crystals or zeolitic fragments in their structure. The use of surfactants allows for the development of the hierarchical catalysts with very narrow pore size distribution. The properties of interzeolite transformation intermediates (ITIs) can be fine-tuned simply by stopping the interconversion at different times.

Table of Contents

1. Experimental Procedures 2
1.1. Acidity by decomposition of isopropylamine in a simultaneous thermal analyzer 2

2. Results and Discussion 2
2.1. Summary of all synthesized samples and their textural and structural characterization 2
2.2. Evolution of the Si/Al ratio of the obtained materials 3
2.3. Analysis of control samples: interzeolite transformation of calcined mesoporous FAU 3
2.4. Ar physisorption at -196 °C at low partial pressures (P/P_0) 4
2.5. UV-Raman spectrum of the parent FAU zeolite 5
2.6. FTIR spectra of solids prepared by different methods of transformation evaluated at different crystallization times 5
2.7. Selected properties and catalytic results of all samples in the Friedel-Crafts alkylation and Claisen-Schmidt condensation reactions 6
2.8. Selected properties and catalytic results in the catalytic cracking of polystyrene 7

References 8

1. Experimental Procedures

1.1. Acidity by decomposition of isopropylamine in a simultaneous thermal analyzer. Sample activation was performed in a simultaneous thermal analyzer (STA) with a heating ramp of 10 °C min^{-1} and 1 L min^{-1} flow of dried air. Ceramic crucibles containing ca. 80 mg of each zeolite were used. Humidity was determined as the mass differences between 50 °C and 550 °C. The system was then cooled to 15 °C before 0.5 mL isopropylamine was added to the sample. The STA was closed and kept at 15 °C for 30 minutes and subsequently slowly evacuated to 1 x 10^{-3} mbar. The samples were heated using two different ramps: (i) 5 °C min^{-1} to 120 °C and (ii) 10 °C min^{-1} to 550 °C. A well-defined endothermic peak is observed in most DSC curves between 280 °C and 410 °C , which is associated to a significant change in mass showed at the TG curves.

Using both curves (DSC-TG), the mass due to the isopropylamine decomposition was determined. The amount of acidic sites could then be determined as follows:

$$Acidity \; = \frac{(\Delta w * (100 - \%w_{lost}))}{100 * W\ zeolite * 56.11\ g/mol} \times 10^{6}\ (\mu\text{mol g}^{-1}) \qquad \text{(S1)}$$

Where Δw is the mass lost by decomposition of isopropylamine in grams, $W_{zeolite}$ is the initial mass of the zeolite n the crucible, $\%w_{lost}$ is the % of humidity in the sample determined in the sample activation.

2. Results and Discussion

2.1. Summary of all synthesized samples and their textural and structural characterization. Samples were prepared by the three interzeolite transformation methods at different treatment times, and intermediate materials were thoroughly characterized. In the case of the fist method (ITI1), the starting zeolite (CBV 720) was hydrothermally treated in the presence of the structural SDA (TEAOH). In the second case (ITI2) a surfactant-templated zeolite with the surfactant (CTAB) incorporated to its structure (uncalcined) was treated with the same SDA. Finally, in the third method (ITI 3), the CBV 720 was treated by using a mixture of the SDA and CTAB. In all cases, the treatment time was extended until the crystalline BEA phase was obtained. Table S1 shows the details of all the samples involved in this study and their characterization.

Table S1. Main properties of all the samples prepared using the three methods at different times.

Sample	Method	Treatment time (d)	Phase	%BEA[a]	Vmicro[b] ($cm^3\ g^{-1}$)	Vmeso[b] ($cm^3\ g^{-1}$)	Vtot[b] ($cm^3\ g^{-1}$)	Recovery yield (%)	Si/Al[c] ratio
**CBV 720**	–	–	FAU	0	0.28	0.18	0.46	–	15.5
**ITI1-0.5**	1	0.5	Am.	7.8	0.03	0.56	0.59	69.9	8.76
**ITI1-0.75**		0.75	Am. + BEA	39	0.08	0.42	0.50	59.4	12.7
**ITI1-1**		1	BEA	98	0.25	0.12	0.37	62.5	14.7
**ITI1-1.5**		1.5	BEA	100	0.25	0.12	0.37	52.1	13.8
**720meso**	–	–	FAU	0	0.15	0.33	0.48	-	16.0
**ITI2-0.5**	2	0.5	Am.	5.0	0	0.65	0.65	80.3	8.07
**ITI2-1**		1	Am. + BEA	49	0.05	0.51	0.56	75.5	10.8
**ITI2-1.5**		1.5	BEA	87	0.08	0.37	0.45	65.5	14.0
**ITI2-2**		2	BEA	94	0.16	0.15	0.31	88.1	12.4
**ITI3-2**	3	2	Am. + BEA	19	0.02	0.41	0.43	75.8	13.2
**ITI3-4**		4	Am. + BEA	26	0.07	0.39	0.46	87.3	13.5
**ITI3-6**		6	Am. + BEA	48	0.13	0.33	0.46	68.3	14.8
**ITI3-8**		8	BEA	71	0.18	0.16	0.34	79.3	15.3
**CP 814E**	–	–	BEA	100	0.22	0.07	0.30	–	12.5[d]
**Al-MCM-41**	–	–	–	–	0	0.80	0.80	–	15.0[e]

[a] Calculated from the XRD analysis using a known amount of graphite as internal standard to normalize the spectra. The most crystalline BEA zeolite obtained after interzeolite transformation is completed, this is, zeolite ITI1-1.5, was defined as 100% BEA and used as reference to calculate the percentage of BEA in the ITIs.

[b] Cumulative pore volumes and pore-size distribution curves were calculated by using the DFT method (NL-DFT adsorption branch model). The total pore volume was obtained at the plateau of the cumulative adsorption pore volume plot at a relative pressure (P/P0) of 0.9. Micropore volume was determined by NL-DFT as the volume adsorbed at pore sizes <2 nm, and the mesopore volume was calculated by subtracting the micropore volume from the total pore volume

[c] Determined by XRF.

[d] As reported by the supplier.

[e] Nominal amount.

2.2. Evolution of the Si/Al ratio of the obtained materials. Figure S1 shows the evolution of the Si/Al ratio of the materials obtained as a function of their crystallinity. Their Si and Al content was determined by X-ray fluorescence (XRF) and the crystallinity was referred to the most crystalline sample (ITI1-1.5, 100% crystalline). Those samples prepared using higher amounts of quaternary amines (ITI3, SDA+CTAB) have Si/Al ratios very similar to the original CBV720, namely 15.5. It is well known that quaternary amines precipitates/avoid the dissolution of silica in basic environments, which helps to keep a high Si/Al ratio and excellent recovery yields.

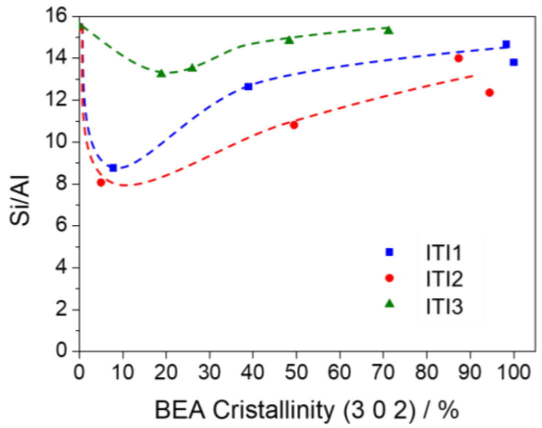

Figure S1. Evolution of the Si/Al ratio related to the BEA crystallinity for samples prepared by the three different methods. Lines are interpolations to guide the eye.

2.3. Analysis of control samples: interzeolite transformation of calcined mesoporous FAU. For comparison purposes, the interzeolite transformation of a mesoporous sample analogous to the one used in ITI2 but after calcination was carried out following the same methodology. The characterization of the obtained materials is presented next: XRD patterns and N_2 isotherms are shown in Figure S2 and TEM images in Figure S3. As can be concluded from the XRD patterns (Figure S2, left), the use of a mesoporous zeolite does not change the kinetics of the interzeolite transformation (ITI1), after a longer induction period. In fact, after 36 h of treatment, ITI1 yields BEA zeolite, 100% crystalline, and if a calcined surfactant-templated FAU is transformed a 98% BEA crystallinity is obtained after 40 h of treatment. The textural properties of the intermediates (Figure S2, center and right) are also very similar irrespectively of using a conventional FAU zeolite (CBV720) or a calcined surfactant-templated FAU zeolite, displaying larger and more irregular mesopores than when CTAB is used during the transformation. Likewise, we did not find any significant differences in the morphology of the intermediates produced using a conventional FAU zeolite (Figure 2, top) or a calcined surfactant-templated FAU zeolite (Figure S3).

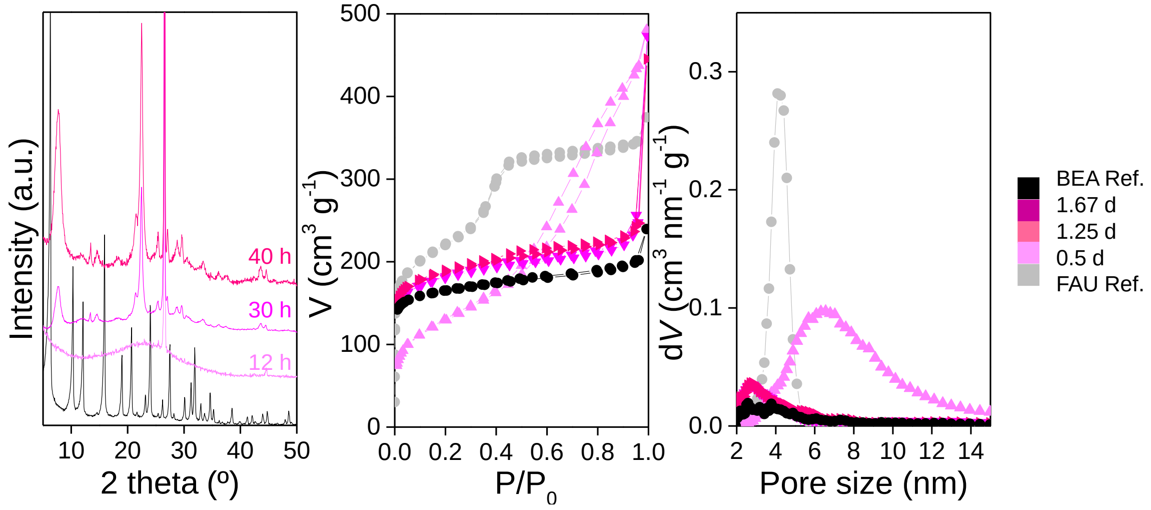

Figure S2. (left) XRD patterns, (center) N_2 physisorption isotherms at -196 C and (right) pore size distribution of the samples prepared at different times of treatment (shown in the legend) using the ITI1 procedure to transform a calcined surfactant-templated FAU zeolite.

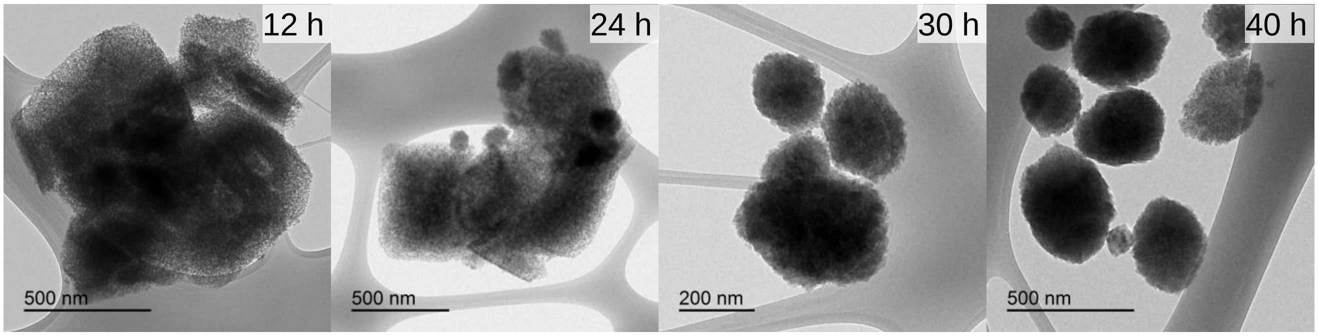

Figure S3. TEM micrographs of samples prepared at different times of treatment (shown in the images) using the ITI1 procedure to transform a calcined surfactant-templated FAU zeolite.

2.4. Ar physisorption at -196 °C at low partial pressures (P/P_0). Figure S4 presents the adsorption profiles of the samples in the low P/P_0 range (10^{-7} – 0.01). The analysis of the adsorption in this region allows studying the evolution of the size and shape of the micropore system in the samples.

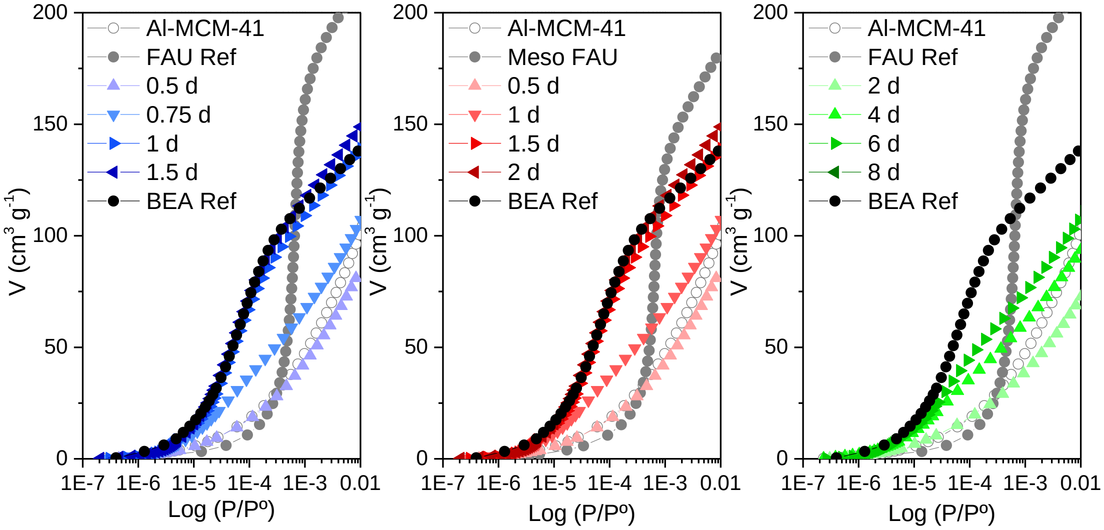

Figure S4. Ar physisorption isotherms at -196 °C of samples prepared by treatment of (left) a parent FAU zeolite with TEAOH, (center) an uncalcined surfactant-templated FAU zeolite with TEAOH and, (right) a parent FAU zeolite with a mixture of CTAB and TEAOH, at different times of treatment (shown in the legend).

2.5. UV-Raman spectrum of the parent FAU zeolite. Figure S5 presents the spectrum of the parent FAU zeolite where the characteristic bands of the FAU crystalline framework can be clearly observed. The peak associated to the vibration of the breathing mode of the 4-membered ring (4R) is found at ca. 508 cm^{-1},[2] a shoulder band due to the breathing vibration mode of the 4R in the double 6-membered rings (D6R) is centered at 490 cm^{-1}, [3] and a third broader band caused by the bending mode of the D6R is located at ca. 300 cm^{-1}.[2,4].

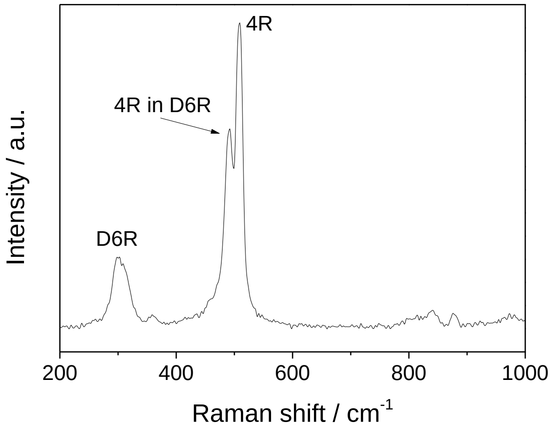

Figure S5. UV-Raman spectrum of the parent FAU zeolite (CBV720).

2.6. FTIR spectra of solids prepared by different methods of transformation evaluated at different crystallization times. Figure S6 presents the FTIR spectra of all the solids prepared using the ITI1 and ITI2 methods. Four sharp peaks at 460, 484, 530, and 613 cm^{-1} are observed in the FAU spectrum, which are due to the internal (460 and 484 cm^{-1}) and external (530 cm^{-1}) T–O–T bending vibration, whereas the peak at 613 cm^{-1} corresponds to the double six-membered ring (D6R) vibration.[5] Intermediate samples, do not show the IR peak associated to the vibration of the D6R, which indicates the loss of the FAU structure. The formation of the BEA structure can be followed by the development of a peak at 575 cm^{-1}, attributed to 5R vibration,[6] which also corresponds with the samples showing the first peaks in the XRD patterns.

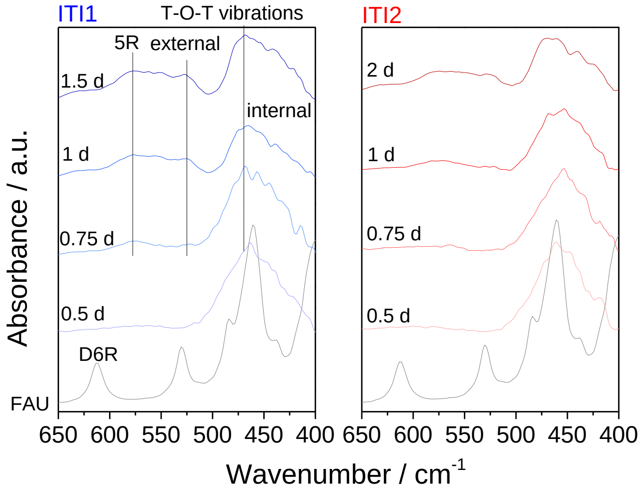

Figure S6. FTIR spectra of samples prepared by method ITI1 and ITI2.

2.7. Selected properties and catalytic results of all samples in the Friedel-Crafts alkylation and Claisen-Schmidt condensation reactions. Table S2 shows the main properties of all the samples involved in this study and their catalytic performance in Friedel-Crafts alkylation and Claisen-Schmidt condensation reactions. Figure S7 presents the % of BEA formed versus acidity and volume of mesopore for every method of transformation.

Table S2. Main properties including crystallinity, acidity, and mesopore volume of all the samples involved in this study and their catalytic performance in Friedel-Crafts alkylation and Claisen-Schmidt condensation reactions.

Sample	Phase	%BEA[a]	Vmeso[b] ($cm^3 g^{-1}$)	Acidity[c] (μmol IPA g^{-1})	Friedel-Crafts			Claisen–Schmidt condensation		
					Conv. (%)	Desv. (%)	TOF (min^{-1})	Conv. (%)	Desv. (%)	TOF (h^{-1})
**CBV 720**	FAU	0	0.18	234	25.3	4.50	0.29	41.4	0.93	6.45
**ITI1-0.5**	Am.	7.8	0.56	136	95.3	1.50	1.87	75.8	2.70	20.31
**ITI1-0.75**	Am. + BEA	39	0.42	289	89.7	0.80	0.83	42.6	1.06	5.24
**ITI1-1**	BEA	98	0.12	512	85.1	1.15	0.44	41.5	2.31	3.03
**ITI1-1.5**	BEA	100	0.12	581	85.6	0.76	0.39	40.7	1.37	2.55
**720meso**	FAU	0	0.33	223[a]	51.3	1.53	0.61	42.0	1.18	6.86
**ITI2-0.5**	Am.	5.0	0.65	240	53.7	0.71	0.60	46.4	2.31	6.15
**ITI2-1**	Am. + BEA	49	0.51	364	92.0	2.90	0.67	33.6	2.51	3.37
**ITI2-1.5**	BEA	87	0.37	365	52.4	3.50	0.38	46.6	1.55	4.65
**ITI2-2**	BEA	94	0.15	485	44.7	4.80	0.25	37.7	2.09	2.83
**ITI3-2**	Am. + BEA	19	0.41	134	98.9	0.57	1.97	56.8	2.72	15.4
**ITI3-4**	Am. + BEA	26	0.39	347	90.6	0.90	0.70	28.6	1.93	2.97
**ITI3-6**	Am. + BEA	48	0.33	n.d.	75.8	5.70	n.d.	28.1	3.37	n.d.
**ITI3-8**	BEA	71	0.16	440	70.7	4.00	0.43	38.4	2.12	3.18
**CP 814E**	BEA	100	0.07	468	60.9	1.54	0.35	31.7	1.66	3.24
**Al-MCM-41**	–	–	0.80	684[d]	65.4	4.67	0.25	28.58	1.97	2.06

[a] Calculated from the XRD analysis using a known amount of graphite as internal standard to normalize the spectra. The most crystalline BEA zeolite obtained after interzeolite transformation is completed, this is, zeolite ITI1-1.5, was defined as 100% BEA and used as reference to calculate the percentage of BEA in the ITIs.

[b] Cumulative pore volumes and pore-size distribution curves were calculated by using the DFT method (NL-DFT adsorption branch model). The total pore volume was obtained at the plateau of the cumulative adsorption pore volume plot at a relative pressure (P/P0) of 0.9. Micropore volume was determined by NL-DFT as the volume adsorbed at pore sizes <2 nm, and the mesopore volume was calculated by subtracting the micropore volume from the total pore volume

[c] Determined by decomposition of isopropylamine in a simultaneous thermal analyzer as described in section 1.1 of the ESI.

[d] Total acid sites (Brønsted and Lewis) in μmol of adsorbed pyridine per gram of zeolite after evacuation at 150 °C. As described in reference [7].

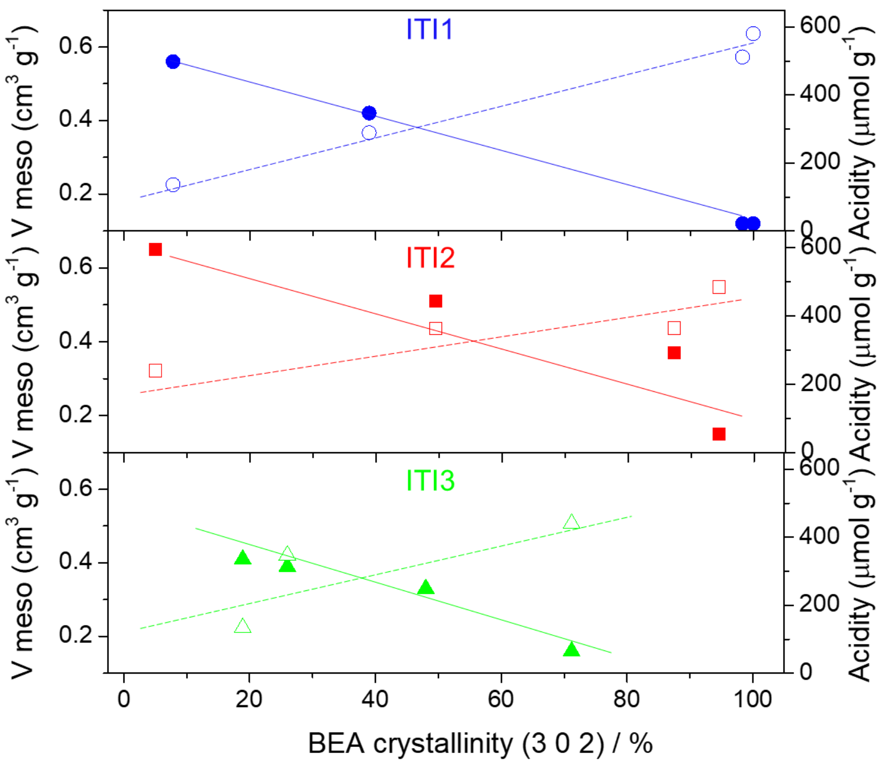

Figure S7. Acidity and volume of mesopore for every method of transformation versus the % of BEA formed.

2.8. Selected properties and catalytic results in the catalytic cracking of polystyrene. Table S3 summarizes the catalytic results of the samples in the cracking of polystyrene. Figure S8 presents the TG and DTG results for every catalytic reaction.

Table S3. Main properties including crystallinity, acidity, and mesopore volume of selected samples involved in this study and their catalytic performance in the cracking of polystyrene.

Sample	Phase	%BEA[a]	Vmeso[b] ($cm^3 g^{-1}$)	Acidity[c] (μmol IPA g^{-1})	T max. (ºC)	ΔT (ºC)	Conv. [e] (%)	TOF [f] (min^{-1})
**CBV 720**	FAU	0	0.18	234	424.1	0	18.65	0.78
**ITI1-0.5**	Am.	7.8	0.56	136	383.7	-40.4	30.66	2.21
**ITI1-0.75**	Am. + BEA	39	0.42	289	385.2	-38.9	29.85	1.02
**ITI1-1**	BEA	98	0.12	512	405.4	-18.7	27.64	1.08
**ITI1-1.5**	BEA	100	0.12	581	396.5	-27.6	25.28	0.43
**720meso**	FAU	0	0.33	223[a]	393.5	-30.5	35.33	1.54
**ITI2-0.5**	Am.	5.0	0.65	240	365.6	-58.5	36.22	1.48
**ITI2-1**	Am. + BEA	49	0.51	364	374.5	-46.3	34.35	0.92
**ITI2-1.5**	BEA	87	0.37	365	407.3	-16.8	25.26	0.68
**ITI2-2**	BEA	94	0.15	485	408.3	-15.8	21.02	0.42
**ITI3-2**	Am. + BEA	19	0.41	134	376.4	-47.7	33.33	2.45
**ITI3-4**	Am. + BEA	26	0.39	347	382.3	-41.8	29.84	0.85
**ITI3-6**	Am. + BEA	48	0.33	n.d.	395.7	-28.4	26.48	n.d.
**ITI3-8**	BEA	71	0.16	440	390.6	-33.5	27.55	0.61
**CP 814E**	BEA	100	0.07	468	413.3	-10.9	13.16	0.27
**Al-MCM-41**	–	–	0.80	684[d]	386.0	-38.1	30.07	0.67

[a] Calculated from the XRD analysis using a known amount of graphite as internal standard to normalize the spectra. The most crystalline BEA zeolite obtained after interzeolite transformation is completed, this is, zeolite ITI1-1.5, was defined as 100% BEA and used as reference to calculate the percentage of BEA in the ITIs.

[b] Cumulative pore volumes and pore-size distribution curves were calculated by using the DFT method (NL-DFT adsorption branch model). The total pore volume was obtained at the plateau of the cumulative adsorption pore volume plot at a relative pressure (P/P0) of 0.9. Micropore volume was determined by NL-DFT as the volume adsorbed at pore sizes <2 nm, and the mesopore volume was calculated by subtracting the micropore volume from the total pore volume.
[c] Determined by decomposition of isopropylamine in a simultaneous thermal analyzer as described in section 1.1 of the ESI.
[d] Total acid sites in μmol of adsorbed pyridine per gram of zeolite after evacuation at 150 °C. As described in reference [7].
[e] Conversion of polystyrene calculated from the amount of weight loss at 350 °C.
[d] Turn over frequency calculated taking into account the conversion of polystyrene at 350 °C.

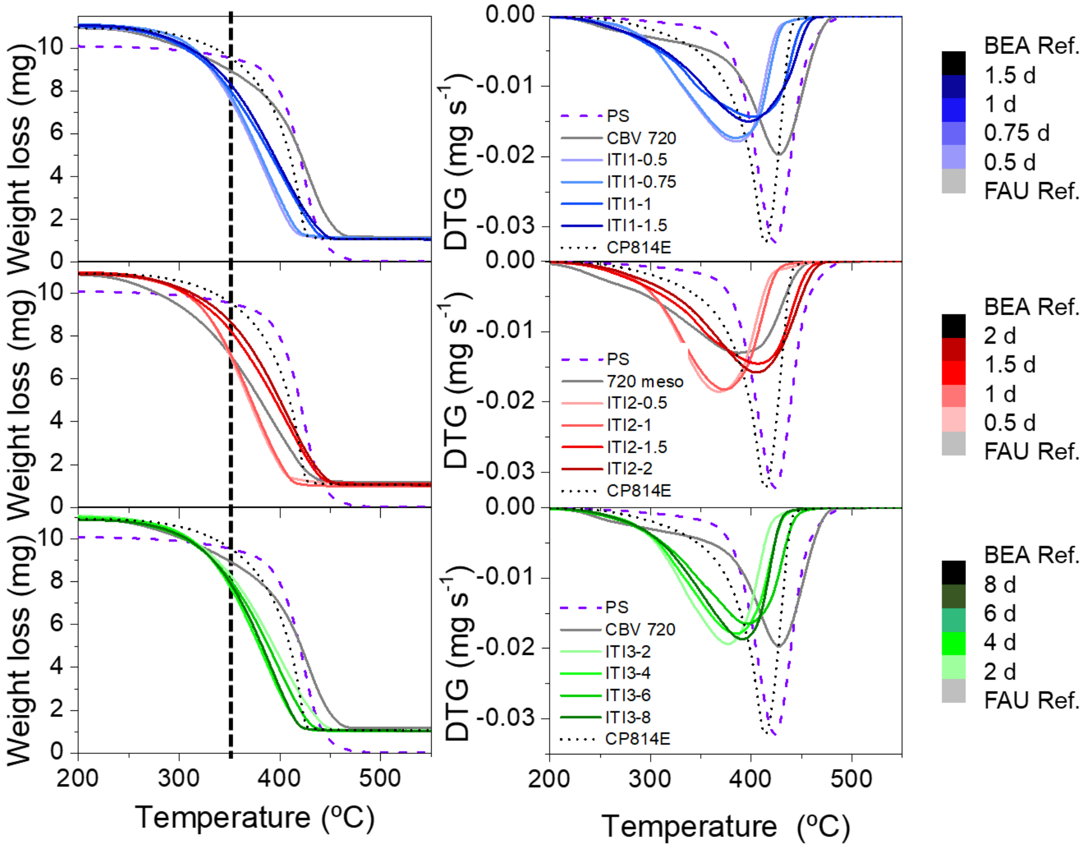

Figure S8. (left) TG and (right) DTG results for catalytic reactions performed with the different samples prepared by every method of transformation. Dotted line marks the weight loss at 350 °C in which the conversion of polystyrene has been calculated.

References

(1) Alhumaidan, F.; Cresswell, D.; Garforth, A. Long-Term Deactivation of Supported Pt Catalysts in the Dehydrogenation of Methylcyclohexane to Toluene. *Ind. Eng. Chem. Res.* **2010**, *49* (20), 9764–9770. https://doi.org/10.1021/ie1013025.

(2) Yu, Y.; Xiong, G.; Li, C.; Xiao, F. S. Characterization of Aluminosilicate Zeolites by UV Raman Spectroscopy. *Microporous Mesoporous Mater.* **2001**, *46* (1), 23–34. https://doi.org/10.1016/S1387-1811(01)00271-2.

(3) Zhang, J.; Chu, Y.; Liu, X.; Xu, H.; Meng, X.; Feng, Z.; Xiao, F. S. Interzeolite Transformation from FAU to CHA and MFI Zeolites Monitored by UV Raman Spectroscopy. *Chinese J. Catal.* **2019**, *40* (12), 1854–1859. https://doi.org/10.1016/S1872-2067(19)63287-0.

(4) Fan, F.; Feng, Z.; Li, G.; Sun, K.; Ying, P.; Li, C. In Situ UV Raman Spectroscopic Studies on the Synthesis Mechanism of Zeolite X. *Chem. - A Eur. J.* **2008**, *14* (17), 5125–5129. https://doi.org/10.1002/chem.200800560.

(5) Halasz, I.; Agarwal, M.; Marcus, B.; Cormier, W. E. Molecular Spectra and Polarity Sieving of Aluminum Deficient Hydrophobic H-Y Zeolites. *Microporous Mesoporous Mater.* **2005**, *84* (1–3), 318–331. https://doi.org/10.1016/j.micromeso.2005.05.040.

(6) Mozgawa, W. The Relation between Structure and Vibrational Spectra of Natural Zeolites. In *Journal of Molecular Structure*; Elsevier, 2001; Vol. 596, pp 129–137. https://doi.org/10.1016/S0022-2860(01)00741-4.

(7) Linares, N.; Cirujano, F. G.; De Vos, D. E.; García-Martínez, J. Surfactant-Templated Zeolites for the Production of Active Pharmaceutical Intermediates. *Chem. Commun.* **2019**, *55* (85). https://doi.org/10.1039/c9cc06696a.
